# Congenital Anhyclosis of Sixteen Joints

**Published:** 1893-10

**Authors:** Samuel E. Milliken

**Affiliations:** Lecturer on Surgery in the New York Polyclinic; New York


For Texas Medical Journal.

### CONGENITAL ANCHYLOSIS OF SIXTEEN JOINTS. ROPORT OF A CASE.

BY SAMUEL E. MILLIKEN, M. D.,
Lecturer on Surgery in the New York Polyclinic.

ANCHYLOSIS of joints may follow any of the inflammatory affections, whether of the synovial membrane, the ends of the bones or the peri articular structures. The case under consideration has been titled congenital, and I think the history and condition will bear me out in that diagnosis. The patient, a female of 10 months, was referred to me by Dr. S. B. Minden, the mother stating that she had accidentally detected a stiffness of the joints of the fingers of both hands when the child was two months of age, but as no pain was complained of, and the health otherwise good, not until now did she consult a physician. My first examination was on June 4, 1893, and was as follows: All the second phalangeal articulations of both hands and feet were almost completely anchylosed, except the thumbs and great toes. Only two of the fingers had any motion at all and that was slight. No unnatural enlargement of the joints could be made out and the movements of the first and third joints were perfect. The fingers and toes were straight with one exception, and that finger varied less than five degrees from the rest. The peculiar feature of the case is that only the second phalangeal articulations should be involved, the thumbs and great toes not being affected. The last may possibly be explained owing to the fact that no middle phalangeal joint exists in these members. I have begun manipulating the joints that manifest any degree of motion with the hope of increasing the same. The case is reported at present because of its uniqueness, as I have not been able to find recorded anything similar.

